# Fungal endocarditis complicating intracranial multifocal hemorrhages: a case report

**DOI:** 10.3389/fcvm.2025.1675873

**Published:** 2025-12-02

**Authors:** Xinyuan Han, Zhijun Huang, Yu Li, Jiuqi Liu

**Affiliations:** 1 Neurological Rehabilitation Department, Shaanxi Provincial Rehabilitation Hospital, Xi'an, China; 2 Xi’an Medical University, Xi'an, China

**Keywords:** case report, fungal endocarditis, mitral valve replacement, intracranial multifocal hemorrhage, antifungal treatment

## Abstract

Fungal endocarditis carries a high mortality rate, with intracranial multifocal hemorrhage representing a severe yet rarely reported complication. We present a case of a 60-year-old female with a history of rheumatic valvular disease who underwent bioprosthetic mitral valve replacement and developed fungal endocarditis with vegetation formation 12 years postoperatively. Following antifungal therapy, the patient underwent redo mitral valve replacement. The postoperative course was complicated by acute subdural hematoma, subarachnoid hemorrhage, and cerebral herniation, necessitating emergent burr hole drainage. Multidisciplinary management achieved hemodynamic stabilization, though with residual neurological deficits including hemiplegia and dysphagia.This case highlights the critical risk of fatal intracranial hemorrhage during the perioperative period of fungal endocarditis, underscoring the need for enhanced monitoring and proactive intervention. The clinical course provides valuable insights for managing such life-threatening complications.

## Introduction

Fungal endocarditis (FE) is a condition associated with high mortality, largely attributable to a spectrum of severe complications (1). In contrast to bacterial endocarditis, fungal vegetations are typically bulky and friable. Embolization of these friable vegetations poses the principal threat, potentially causing ischemic stroke or forming mycotic aneurysms (MAs) that are prone to rupture and may culminate in fatal intracranial hemorrhage (ICH). The destructive potential of fungal pathogens can also lead to extensive valvular damage, precipitating intractable heart failure that frequently necessitates emergent surgical intervention (2). Consequently, early detection of these complications coupled with the timely initiation of targeted therapy are pivotal for improving clinical outcomes. This article elucidates a distinctive case to offer valuable insights into the diagnosis and management of these complex infections when complicated by ICH.

## Case description

**Figure 1:**
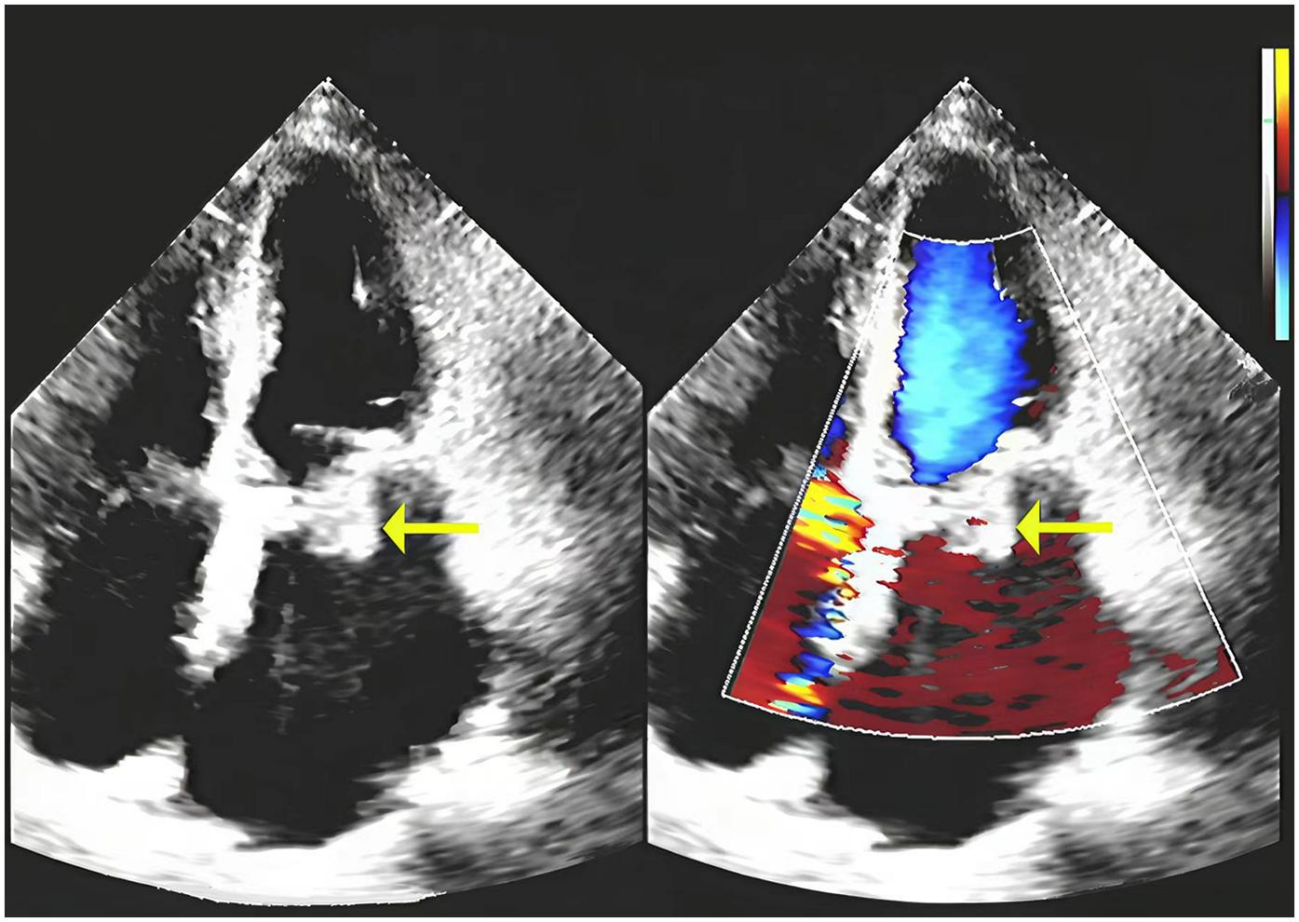
Transthoracic echocardiography (left panel) and color Doppler image (right panel) demonstrate a vegetation on the mitral valve (yellow arrow).

This case report describes a 60-year-old female patient with a history of rheumatic valvular disease complicated by mitral regurgitation and atrial fibrillation, who underwent bioprosthetic mitral valve replacement (MVR) 12 years prior. Postoperatively, she maintained long-term warfarin therapy (6 mg daily). The current admission was prompted by a 10-day history of worsening fever (38.2 ℃), palpitations, chest tightness, and dyspnea. Cardiac auscultation revealed a holosystolic blowing murmur at the apex, radiating to the left axilla. Laboratory investigations showed marked elevations in C-reactive protein (CRP 25.03 mg/L) and procalcitonin (PCT 5.39 ng/mL). Transthoracic echocardiography (TTE) demonstrated structural deterioration of the mitral bioprosthesis, featuring a 2.2 cm × 1.6 cm vegetation (Figure 1), an elevated mean transvalvular gradient of 34 mmHg, and severe tricuspid regurgitation. Preliminary assessment yielded the following diagnoses: suspected infective endocarditis, acute decompensation of chronic heart failure (NYHA class IV), rheumatic heart disease (status post bioprosthetic MVR), and permanent atrial fibrillation (CHADS₂-VASc score 4). Despite the initiation of intravenous piperacillin-tazobactam (4.5 g every 8 h) following blood culture collection, the patient's clinical condition showed no substantial improvement.

Following 54 h of incubation in the BD BACTEC® system (Becton Dickinson, USA), blood cultures yielded positivity for yeast-like spores. Subsequent analysis via MALDI-TOF MS (Bruker, USA) confirmed the isolate as *Wickerhamomyces anomalus*. Antifungal susceptibility testing, performed by broth microdilution, determined the minimum inhibitory concentrations (MICs) against this pathogen. The resulting MIC profile demonstrated susceptibility to amphotericin B (0.25 mg/L), caspofungin (0.03 mg/L), fluconazole (4 mg/L), and posaconazole (0.5 mg/L). On the subsequent day, she developed rapid clinical deterioration progressing to cardiogenic shock, with a recorded blood pressure of 73/41 mmHg. A multidisciplinary consensus from our institutional heart team, incorporating neurosurgeons and anesthesiologists, judged the patient's profound hemodynamic instability to confer an unacceptably high mortality risk associated with intrahospital transport for cranial computed tomography (CT). Thereupon, after comprehensive family consultation and procurement of informed consent, the clinical team opted to forgo preoperative neuroimaging and proceed directly with emergent valve replacement as a critical intervention.

**Figure 2:**
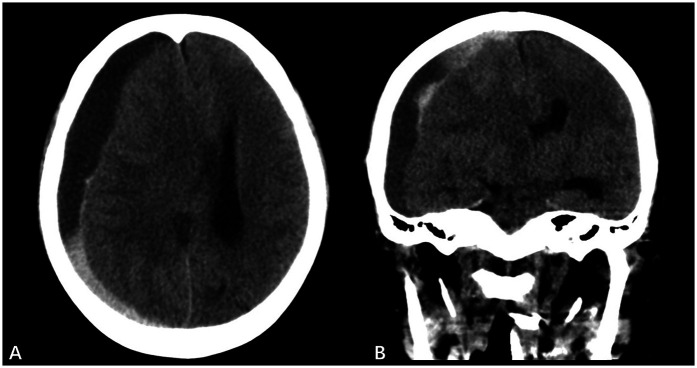
Preoperative CT scans **(A)** and **(B)** demonstrate: right fronto-temporo-parieto-occipital subdural hematoma, brain herniation, Minor subarachnoid hemorrhage, midline shift.

The patient underwent redo bioprosthetic MVR combined with tricuspid valvuloplasty. Surgical exploration identified a 2.2 cm × 1.6 cm vegetation adherent to the prosthetic mitral valve, which was associated with leaflet prolapse, critical stenosis of the valvular orifice, and concomitant tricuspid regurgitation. Valve intervention involved replacing the native mitral valve with a 25 mm bioprosthesis (Edwards Lifesciences, Irvine, CA) and implanting a 28 mm annuloplasty ring on the tricuspid valve. Histopathological assessment of the resected vegetation, with confirmation by Grocott-Gomori's Methenamine Silver (GMS) staining, established the diagnosis of fungal infection. Perioperative anticoagulation was switched from warfarin to enoxaparin (1 mg/kg twice daily). Postoperatively, she developed coma (GCS 6) with anisocoria (right pupil dilated and nonreactive to light). Emergency head CT revealed a right-sided subdural hematoma (SDH) with transtentorial herniation and minor subarachnoid hemorrhage (SAH) (Figures 2A,B). Burr hole evacuation and drainage were performed immediately, and all anticoagulants were discontinued (detailed in Table 1). One week later, although her consciousness partially improved (GCS 10), she still sustained hypotension (85/46 mmHg) and high-grade fever (39.5 °C).

**Table 1:** Perioperative anticoagulant management in FE and adjustment timeline for treatment after ICH.

Timeline	Key events	Therapeutic regimen and decisions	INR
Initial admission	High clinical suspicion of IE	Warfarin dosage adjusted from 6 mg/day to 5 mg/day	3.12
Hospital Day 3/preoperative	Clinical confirmation of FE	Warfarin discontinued, switched to enoxaparin 1 mg/kg twice daily	2.96
Hospital Day 4/Day of surgery	Emergency valve replacement due to worsening heart failure	Enoxaparin discontinued preoperatively; LMWH initiated postoperatively	Intraoperative ACT monitoring
Postoperative Day 1	Patient developed sudden ICH	All anticoagulants discontinued; emergency burr hole drainage performed	—
Within first 4 postoperative weeks	Transferred back to ICU with persistent coma and hypotension	Anticoagulation withheld; close monitoring of surgical site and intracranial hemorrhage	1.75
After 4 postoperative weeks	Cranial CT showed no new hemorrhage; MDT evaluation recommended cautious resumption of anticoagulation	Prophylactic-dose LMWH (enoxaparin 40 mg once daily) restarted	1.03
3 days after anticoagulation resumption	Clinical condition remained stable	Oral warfarin 5 mg/day restarted, overlapping with LMWH	1.24
7 days after anticoagulation resumption	Warfarin therapeutic effect achieved	LMWH discontinued after INR remained within target range (2.0-3.0) for two consecutive days; lifelong warfarin 5 mg/day prescribed	2.06

IE, infective endocarditis; FE, fungal endocarditis; ICH, intracranial hemorrhage; LMWH, low molecular weight heparin; INR, international standardized ratio; ACT, activated clotting time; ICU, Intensive Care Unit; MDT, multidisciplinary team.

**Figure 3:**
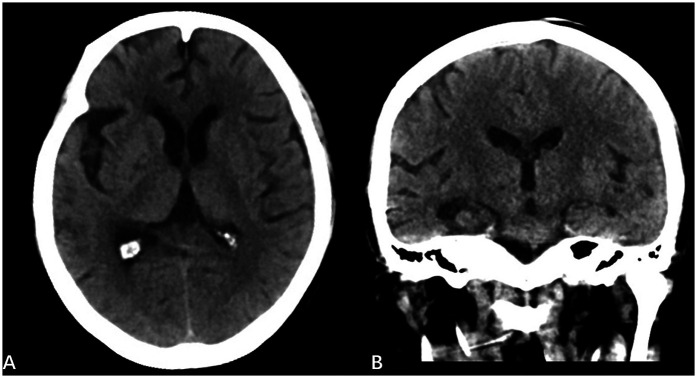
Postoperative CT scans **(A)** and **(B)** show significant reduction in: right fronto-temporo-parieto-occipital subdural hematoma,subarachnoid hemorrhage, compared to preoperative images.

Subsequent laboratory investigations demonstrated severe systemic infection manifesting as leukocytosis (WBC 18.2 × 10^9^/L) with neutrophil predominance (94.5%), accompanied by markedly elevated inflammatory markers including CRP 102.7 mg/L, PCT 58.7 ng/mL, interleukin-6 (IL-6 1,013.9 pg/mL), and (1,3)-β-D-glucan (221.37 pg/mL). Microbiological confirmation was achieved through isolation of *Wickerhamomyces anomalus* from both blood cultures and valvular vegetation specimens, establishing a definitive diagnosis of fungal endocarditis complicated by septic shock. Despite 14 days of combination antifungal therapy comprising sequential intravenous fluconazole (4 mg/kg/day for 7 days) and caspofungin (1 mg/kg/day for 7 days) alongside broad-spectrum antibiotics, the treatment proved ineffective in a 50 kg patient, as indicated by persistent fever and rising (1,3)-β-D-glucan levels to 551.8 pg/mL. Following this failure, the antimicrobial strategy was modified to incorporate intravenous liposomal amphotericin B (4 mg/kg/day) plus caspofungin (1 mg/kg/day), augmented by hydrocortisone and continuous renal replacement therapy (CRRT) for cytokine storm management. After one week of this intensified regimen, conventional infection parameters (WBC, PCT, CRP, IL-6) had normalized; however, the (1,3)-β-D-glucan level remained persistently elevated. A subsequent therapeutic escalation to high-dose liposomal amphotericin B (5 mg/kg/day) combined with enteral posaconazole suspension (400 mg twice daily) resulted in dramatic clinical improvement within seven days, characterized by defervescence, restored consciousness (GCS improvement to 15), and normalization of all infection markers including (1,3)-β-D-glucan. Therapeutic drug monitoring (TDM) confirmed a posaconazole trough concentration of 1.6 μg/mL. Concurrent neuroimaging revealed substantial resolution of ICH (Figures 3A,B).

The patient exhibited residual neurological deficits including right-sided limb muscle strength grade IV, left upper extremity strength grade III^+^, left lower extremity strength grade II, dysarthria, and dysphagia requiring nasogastric tube feeding. Completion of the six-week intravenous liposomal amphotericin B regimen initiated a transition to antifungal maintenance with posaconazole suspension (400 mg twice daily) delivered via nasogastric tube. Following 3 months of systematic rehabilitation, significant functional improvement was achieved with complete resolution of speech impairment, improvement of limb muscle strength to grade IV bilaterally (left lower extremity grade IV^−^), ability to ambulate with a cane, and enhanced swallowing function (Wada drinking test grade 2), supported conversion to long-term suppression therapy using oral posaconazole delayed-release tablets (300 mg daily). We recommended continuing this antifungal prophylaxis for at least two years, supplemented by a structured surveillance protocol comprising quarterly posaconazole TDM, monthly hepatic function panels, and serial TTE at three-month intervals. Additionally, comprehensive multidisciplinary evaluations at cardiac surgery clinics were scheduled every 3–6 months to monitor overall health status and neurological sequelae. Following hospital release during a period of clinical stability, the patient participated in regular follow-up assessments. Neurological status documented at discharge revealed a modified Rankin Scale score of 3, which subsequently improved to grade 2 over the subsequent three-month period. Follow-up echocardiographic evaluation confirmed normal positioning, mobility, and echotexture of the mitral bioprosthesis with only trace tricuspid regurgitation.

## Discussion

This case of FE complicated by secondary ICH underscores the diagnostic and therapeutic challenges inherent to this condition. The isolation of *Wickerhamomyces anomalus*—an uncommon pathogen with pronounced biofilm-forming capacity and prosthetic valve affinity—significantly compounded the treatment complexity (3). Contemporary data indicate that while FE accounts for merely 1%–3% of all infective endocarditis cases, it carries a staggering mortality exceeding 70% (1). ICH as the initial presentation, though rare, portends particularly dismal outcomes (2). In the present case, subdural hematoma with subsequent herniation likely originated from either ruptured mycotic aneurysms or septic emboli-induced vasculitic necrosis. Of critical importance, the incidence of mycotic aneurysms in FE substantially surpasses that observed in bacterial endocarditis (4), compounded by the suboptimal sensitivity (57%) of Computed Tomography Angiography (CTA) in detecting aneurysms <5 mm (2). This pathophysiological continuum finds validation in reported cases of *Aspergillus flavus* endocarditis with multifocal cerebral hemorrhage, where hyphal embolization precipitates distal vasculitis and pseudoaneurysm formation (5).

The standard diagnostic protocol for suspected infective endocarditis with substantial vegetations comprises comprehensive preoperative assessment, including neuroimaging (CTA/MRA) and transesophageal echocardiography (1, 2). Nevertheless, this patient's clinical presentation with hemodynamic compromise precluded the safe completion of these essential investigations. These diagnostic limitations consequently impeded the prompt recognition of FE and associated complications.

The fatal ICH following perioperative transition from warfarin to enoxaparin in this case exemplifies the critical challenge of anticoagulation management in prosthetic valve recipients. The exceptionally large fungal vegetation (2.2 × 1.6 cm in this instance) demonstrates heightened embolic potential, with FE carrying substantially greater embolism risk compared to bacterial counterparts (2). This case reveals the dual jeopardy of anticoagulation in such scenarios: the infectious process itself induces vasculitis and coagulopathy, while cardiothoracic surgery further disrupts hemostatic balance. Current ESC 2023 guidelines emphasize a cautious approach, recommending gradual reintroduction of anticoagulation after a 4-week stabilization period in ICH cases, with oral anticoagulants preferred due to their superior safety profile regarding rebleeding risk (2). While reinitiating anticoagulation remains imperative for preventing lethal thromboembolism, it inherently elevates ICH risk. Navigating this therapeutic dilemma requires meticulous risk stratification through multidisciplinary team (MDT) evaluation, followed by nuanced calibration of timing and initial dosing to optimize this delicate risk-benefit equilibrium (6). Notably, the absence of valvular thrombosis despite prolonged anticoagulation interruption in our patient may reflect both the complete surgical debridement of infected tissues and the efficacy of prompt, aggressive antifungal therapy, providing important clinical implications for personalized therapeutic strategies in high-risk populations.

This clinical scenario supports expediting surgical intervention as the preferred approach for patients experiencing critical hemodynamic instability (2), even when accounting for indeterminate neurological sequelae. The inherent fragility of pre-existing mycotic aneurysms predisposes them to rupture despite meticulous perioperative anticoagulation management, potentially culminating in postoperative ICH (4). Such outcomes represent not guideline non-adherence but rather necessary risk stratification between imminent circulatory failure and potential neurological compromise.

The antifungal regimen in this case underwent stepwise escalation from fluconazole → caspofungin → liposomal amphotericin B+ caspofungin → ultimately successful control with liposomal amphotericin B+ posaconazole. This therapeutic optimization was necessitated by: (i) potential intrinsic azole resistance in *Wickerhamomyces anomalus* compounded by biofilm-mediated drug penetration barriers (3); and (ii) posaconazole's broader spectrum and superior efficacy/safety profile against fungal pathogens compared to fluconazole (7). Notably, the paradoxical elevation in serum *β*-D-glucan levels during initial therapy (221.37 → 551.8 pg/mL) likely represented massive fungal lysis rather than treatment failure—a documented phenomenon in effectively treated patients (1). Recent precision medicine research by Cusato et al. demonstrates that pharmacogenomics-guided posaconazole dosing achieves target concentrations several-fold more frequently (8), offering novel strategies for drug-resistant infections through optimized therapeutic drug monitoring.

The patient's secondary septic shock was characterized by markedly elevated IL-6 (1,013.9 pg/mL), signifying cytokine storm. Management with CRRT for inflammatory mediator clearance combined with short-term hydrocortisone (≤200 mg/day) aligns with contemporary septic shock principles, with studies demonstrating CRRT's capacity to reduce vasopressor requirements (9). Regarding neurological intervention, emergent burr hole decompression provided definitive survival benefit in this herniating patient with GCS <8 (10). However, optimal cardiac surgery timing for FE with ICH remains contentious: a 2021 Cureus case report supports staged intervention with initial neurological stabilization followed by valve surgery after 2 weeks (11), while the European Heart Journal recommends postponement until 3–4 weeks post-hemorrhage (2).

This successful outcome relied on highly coordinated multidisciplinary collaboration encompassing cardiac surgery, neurosurgery, critical care, and rehabilitation medicine. Evidence confirms that MDT approaches positively impact outcomes and reduce mortality in FE (12). Lifelong posaconazole suppression post-discharge remains imperative due to persistent prosthetic valve infection risk (1). Significant neurological recovery (muscle strength grade IV, modified Wada water drinking test grade 2, and modified Rankin Scale score of 2) during follow-up underscores the efficacy of early rehabilitation for survivors of mycotic aneurysms.

## Data Availability

The raw data supporting the conclusions of this article will be made available by the authors, without undue reservation.
